# An Immeasurable Crisis? A Criticism of the Millennium Development Goals and Why They Cannot Be Measured

**DOI:** 10.1371/journal.pmed.0020318

**Published:** 2005-09-13

**Authors:** Amir Attaran

## Abstract

Attaran argues that five years into the Millenium Development Goals project, problems with measurement mean that often we cannot know if true progress towards these goals is occurring.

In September 2000, 147 heads of state met at the United Nations (UN) headquarters—the largest such gathering ever—to resolve action on the most pressing problems of humanity and nature [1]. To underscore their commitment, they set numerical targets and deadlines to measure performance. These are the Millennium Development Goals (MDGs), and they span a large range of topics, including poverty, infectious disease, education, and gender equality (Box 1).

**Box 1. The MDGs and Targets**

By the year 2015, UN member states have pledged to meet eight goals; each goal subsumes one or more targets, as reproduced verbatim here (quoted from [40]). Details of the targets subsumed by goal eight and the various indicators for all the goals or targets can be found in [40,41].

**Goal 1: Eradicate extreme poverty and hunger **

• Reduce by half the proportion of people living on less than a dollar a day

• Reduce by half the proportion of people who suffer from hunger

**Goal 2: Achieve universal primary education **

• Ensure that all boys and girls complete a full course of primary schooling

**Goal 3: Promote gender equality and empower women **

• Eliminate gender disparity in primary and secondary education preferably by 2005, and at all levels by 2015

**Goal 4: Reduce child mortality **

• Reduce by two thirds the mortality rate among children under five

**Goal 5: Improve maternal health **

• Reduce by three quarters the maternal mortality ratio

**Goal 6: Combat HIV/AIDS, malaria, and other diseases **

• Halt and begin to reverse the spread of HIV/AIDS

• Halt and begin to reverse the incidence of malaria and other major diseases

**Goal 7: Ensure environmental sustainability **

• Integrate the principles of sustainable development into country policies and programmes; reverse loss of environmental resources

• Reduce by half the proportion of people without sustainable access to safe drinking water

• Achieve significant improvement in lives of at least 100 million slum dwellers, by 2020

**Goal 8: Develop a global partnership for development **

This September, the heads of state will gather again for the Millennium +5 Summit to assess the five-year progress of the MDGs. They will find that the MDGs have become all-important, not just within the UN, but also as the zeitgeist of the global development enterprise. As Professor Jeffrey Sachs, Director of the UN's Millennium Project, has declared, “To the extent that there are any international goals, they are the Millennium Development Goals”[2].

But is it wise to elevate the MDGs to the pedestal where they now sit? Could it be, despite an appearance of firm targets, deadlines, and focused urgency, that the MDGs are actually imprecise and possibly ineffective agents for development progress?

In this article, I argue that many of the most important MDGs, including those to reduce malaria, maternal mortality, or tuberculosis (TB), suffer from a worrying lack of scientifically valid data. While progress on each of these goals is portrayed in time-limited and measurable terms, often the subject matter is so immeasurable, or the measurements are so inadequate, that one cannot know the baseline condition before the MDGs, or know if the desired trend of improvement is actually occurring. Although UN scientists know about these troubles, the necessary corrective steps are being held up by political interference, including by the organisation's senior leadership, who have ordered delays to amendments that could repair the MDGs [3]. In short, five years into the MDG project, in too many cases, one cannot know if true progress towards these very important goals is occurring. Often, one has to guess.

## The MDGs and Principles of Measurement

What makes the MDGs attractive is their concreteness. For example, the MDG to eradicate extreme poverty subsumes a “target” to “halve, between 1990 and 2015, the proportion of people whose income is less than $1 a day”, which in turn subsumes “indicators”, one of which is to measure income based on purchasing power.

Knowing that, worldwide, 28% of people in 1990 had purchasing power below $1 a day gives rise to a benchmark: that in 2015, fewer than 14% of people should be so destitute [4,5]. Currently, East Asia is on track; sub-Saharan Africa is not [6]. Such definitive statements about the benchmark or the trend are possible because non-stop effort goes into measuring incomes and prices—the UN, governments, and businesses all do it—so there are sufficient and reliable data.

It is harder to get sufficient and reliable data for the health MDGs. Even the most basic life indicators, such as births and deaths, are not directly registered in the poorest countries. Within this decade, only one African country (Mauritius) registers such events according to UN standards [7]. Without reliable vital registration systems to track even the existence of births or deaths, naturally the data for the medical circumstances of those births or deaths—or the lives in between—are unreliable.

Accordingly, most of the available data on the health MDGs come from methods of estimation, censuses, specialised household surveys, or all of these together.

There are many—too many—household surveys. In the public-health field, the best known are the Demographic and Health Survey (DHS) and the Multiple Indicator Cluster Survey (MICS), funded mainly by the United States and United Nations Children's Fund (UNICEF), respectively [8]. In addition to those household surveys, the Centers for Disease Control and Prevention, the World Health Organization (WHO), the United Nations Population Fund, the World Bank, and other organisations contribute surveys, making a rich alphabet soup—RHS, WHS, CWIQ, LSMS, PAPFAM, and so on. The proliferation is so excessive that there is now an International Household Survey Network, the rationale for which reads:

*Donor's [sic] support is not always appropriately coordinated. There are many examples of duplicated or conflicting data collection activities. This lack of coordination does not only causes [sic] a huge waste of funds, it also put [sic] a high burden on national statistics offices. In the past few years, significant progress has been made to identify synergies among different survey programs or to develop common questionnaire modules, and to conduct joint data collection activities. But there is certainly room for much more cooperation. [9]*

All of this is true, but even within the UN, different agencies jostle counterproductively for data. For example, in 2002, the WHO launched a new World Health Survey in over 70 countries to compete with the longer-running DHS and MICS [10]. Justified as a “sound basis for evaluating progress towards the millennium development goals”, instead the WHO's new survey tied up the few qualified statistical staff in the poorest countries [11]. Three years later (at the time of going to press), the new project has yet to publish a single dataset. (Ironically, the WHO has since created a new project called the Health Metrics Network, for “reducing overlap and duplication” caused by a “plethora of separate and often overlapping [data] systems” [12]. One cannot yet say whether the Health Metrics Network will succeed at this important goal, or add a further layer to the problem.)

Figure 1 shows the number of reported DHS and MICS surveys since 1990, which is the most common MDG baseline year. To generalise, most countries have had two or three such surveys, each gathering data on perhaps 5,000–10,000 households. Together with other surveys or national censuses, DHS and MICS are the backbone of measuring progress on the MDG health indicators.

**Figure 1 pmed-0020318-g001:**
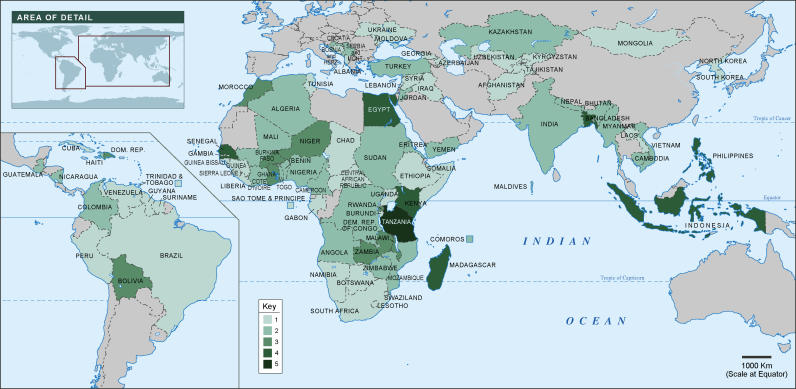
Map of DHS and MICS Surveys The map shows the number of DHS and MICS surveys by country, 1990–2005, according to completed reports made available to the public in June 2005. These reports are top-level summaries of the underlying micro-level survey data. Note, however, that UNICEF has not publicly disclosed micro-level data for 13 countries (Afghanistan, Algeria, Botswana, Cambodia, Cuba, Georgia, India, the Maldives, Somalia, Syria, Tunisia, Ukraine, and Federal Republic of Yugoslavia), making independent verification of those reports impossible (see http://www.childinfo.org; http://www.measuredhs.com/). (Illustration: Bang Wong, www.clearscience.info)

Yet household surveys are serviceable but crude tools. Even with a simple question, such as about a child's birth weight, people's answers only roughly approximate the truth, as would be measured by weighing on a scale [13]. Other survey questions are so technical that no layperson can answer them accurately. MICS, for example, asks parents if their child's anti-malaria bed net was “ever treated with a product to kill mosquitoes”: an accurate answer depends on the type, dose, and date of insecticide treatment, and whether the local mosquito species carry insecticide resistance genes [14]. Because household surveys do not announce these or other sources of error, one can easily have false confidence in them. For example, many MICS survey reports present their findings as single-point estimates, without any of the usual qualifiers of data inaccuracy or quality, such as statistical confidence intervals or significance tests (see India's report for example; [15]).

In short, there are many sources of data on the MDGs. When those sources suffice to reveal statistically significant trends in the MDGs, then all is well, and it is possible to make conclusive statements: that the MDGs are being met, or that the MDGs are being missed. But, as the case studies below illustrate, such certainty is highly elusive.

## Malaria

MDG 6, Target 8, pledges to “have halted by 2015 and begun to reverse the incidence of malaria”. The malaria MDG overlaps with a somewhat earlier (1998) WHO-led goal known as Roll Back Malaria (RBM), which aims “to halve malaria-associated mortality by 2010 and again by 2015” [16]. Even though the MDG and the RBM goal are only quasi-consistent with one another, the UN allows them to coexist, and UN communications often mention both [16]. Accordingly, both are discussed here.

Yet with double attention on malaria, and the head start afforded by RBM, the UN still is unable to make an official pronouncement on the progress of its malaria goals. The WHO and UNICEF write that it is “too soon to determine whether the global burden of malaria”, meaning both incidence and mortality, “has increased or decreased since 2000” [16].

Too soon? RBM is in its seventh year, and past the halfway mark of its 2010 deadline. The only two possible reasons not to know if malaria has increased or decreased are that the UN either (i) did not encourage timely measurements or (ii) chose indicators—malaria incidence and mortality—that are essentially immeasurable.

Actually, both are true. What follows is a cautionary history.

In 2002, the British government commissioned an independent evaluation of the UN's malaria efforts. It did so because it was the largest financier of RBM, and because of a perception that there was insufficient alignment between the efforts of the UN agencies and malarious countries. On the subject of measuring progress, the evaluators wrote: [Fig pmed-0020318-g001]

*The main problem affecting…data collection efforts…has been that an overly complex and insufficiently prescriptive approach has been taken. There has been a failure to clearly define goals and priorities of the [measurement] strategy at the global and regional levels....Too many indicators are proposed. Too many sources of data are suggested. Insufficient guidance is given to countries on data collection and methodology….Some countries are measuring one thing, some countries are measuring another….In some cases, data are being collected without any systematic and scientific sampling methodology, and so are essentially meaningless and impossible to interpret. [17]*

This unsparing criticism points to two problems, which although they pertain to RBM, often apply with equal force to the malaria and other MDGs. The first problem concerns the lack of a baseline: it is impossible to retrospectively measure worldwide (or regional, or national) malaria incidence and mortality existing at the inception of the RBM goal or the MDG, when the data from that era are universally acknowledged to be poor [18]. Without knowing the original condition, it is futile to stipulate either “to halve” malaria mortality by 2010 or “to halt” malaria incidence by 2015. Such words have no meaning where the baseline is mysterious.

The second problem concerns the unsuitability of the indicators: both malaria incidence and mortality are so crudely measured by household surveys and most countries' health records that, essentially, they are immeasurable. The UN's malaria monitoring group agrees, writing that “malaria-specific mortality should not be monitored routinely, as this can not be measured easily in malaria-endemic Africa” [19]. Yet the UN often ignores such warnings, even when they are timely, explicit, and the opinions of its own scientists. It was only two months after WHO scientists wrote that “it will not, in general, be possible to measure the overall incidence rate of malaria” that the UN chose the incidence rate as the mainstay of the malaria MDG [20].

The legacy of unfortunate decisions now leaves malaria risk mapping as the only feasible way to estimate (not measure) malaria incidence and mortality. The principle is to superimpose a map of a population onto a map of malaria intensity, although, in practice, the limitations include malaria maps from the 1960s and too few demographic surveillance sites to accurately measure and calibrate incidence and mortality risks [21,22]. The WHO has been slow to use risk mapping, probably because it fears public criticism when, inevitably, the current estimates of malaria severity must be revised upward [23,24].

Accordingly, years after the withering external evaluation, the UN neither has achieved convincing measurement or estimation of malaria incidence and mortality, nor has it abandoned those as the key indicators of progress. Both the RBM goal and the malaria MDG are today immeasurable.

## Maternal Mortality

MDG 5, Target 6, pledges to “reduce by three quarters, between 1990 and 2015, the maternal mortality ratio” [1]. As such, this MDG target echoes a 1994 UN goal set at the Cairo Conference on Population and Development to halve maternal mortality by 2000, and again by 2015 [25].

The UN Millennium Project reports that at about 530,000 deaths annually, “overall levels of maternal mortality are believed to have remained unchanged” in the last 15 years [26]. Both the number of such deaths and the number of births are used to calculate the maternal mortality ratio (MMR; the number of women dying through complications of pregnancy and delivery per 100,000 live births). However, it is exactly in the poorest countries where the maternal mortality problem is severest that the data about deaths and births are least satisfactory. Vital registration would help, but few developing countries, accounting for 24% of the world's live births, have complete data [7]. Directly measuring MMR in the whole population is not today an option.

Therefore MMR must be estimated. The current method is crude, and uses regression modelling based on partial vital registration, censuses, household surveys, and other inputs [27]. The outputs are a point estimate for MMR in each geographic region, surrounded by an educated guess (not the same as a valid statistical confidence interval) of the lower and upper range in which the point estimate could lie.

Accordingly, the most recent (2000) published estimate for MMR worldwide is 400 maternal deaths per 100,000 births, within an unscientific, best-guess range of perhaps 210 (low) to 620 (high) [28]. Estimates for the MDG baseline year (1990) are similarly vague [29].

Without a statistically robust estimate for MMR in the baseline year, or in later years, nobody knows whether worldwide MMR has increased or decreased since 1990, other than in a “handful of countries” [26]. The limitations of current estimation techniques are so profound that UNICEF and WHO scientists warn that “it would be inappropriate to compare the 2000 estimates with those for 1990…and draw conclusions about trends” [28].

Thus, 11 years after the Cairo Conference first set an explicit target to reduce MMR by 75%, the UN neither has achieved measurement of MMR, nor has it heeded the warnings of its own scientists that MMR is basically immeasurable. The MDG carries that mistaken goal forward to 2015, and the impossibility of measuring and demonstrating success is certainly preordained.

## Tuberculosis

MDG 6, Target 8, pledges to “have halted by 2015 and begun to reverse the incidence of…major diseases”, which the UN has interpreted to include TB [1]. The provenance of the TB MDG is it neither reiterates an earlier (1991) goal, nor is it obviously a purposeful improvement [30].

As with malaria, measuring TB incidence is notoriously difficult. It requires counting the annual number of new patients with TB disease (i.e., not just new TB infections). Currently, no country measures TB incidence regularly, as the MDG target stipulates [31].

Fortunately, the MDG indicators provide for some simpler alternatives: TB disease prevalence and deaths (Indicator 23), and the proportion of TB disease cases detected and cured using a WHO-recommended treatment called “directly observed therapy—short course” (DOTS; Indicator 24). The TB prevalence and case detection indicators are directly measurable, but, ironically, the WHO does not actually measure them. Instead, it uses a unique, arguably outdated estimation method.

Nobody can say with scientific confidence what the actual trends for TB are.

In the WHO's method, the only true measurement is the number of new, sputum-positive TB cases that are detected and notified to the authorities for treatment with DOTS. To estimate the case detection rate, the WHO divides that number of notified TB cases (the numerator) by an estimate of at-large case incidence (the denominator) [32]. Further, the WHO obtains case incidence from “an independent estimate of the case detection rate” [33]. In effect, the WHO's two estimates are circular and lack definite meaning, for each estimate draws upon the other estimate. Further, the WHO bases this estimation process on inputs that are not always rigorous, and the inputted data are often obtained from collective opinion rather than measurement [33].

Accordingly, it is impossible to state the actual trends in TB disease with any degree of statistical confidence. The WHO's best guess is that its estimates “typically range from −20% to [+]40%” in accuracy [32].

Others have criticised the circular estimation technique. The WHO's former director for evidence argues that “essentially no empirical basis exists to assess the trend in case detection in regions where tuberculosis is most prevalent, including sub-Saharan Africa” [34]. He calls the WHO's trend estimates “serial guessing” [34]. Certainly, the WHO's leading assumption (known as the “Styblo rule” [35]) has infrequently been tested in Africa, where TB is accelerated by an unparalleled HIV/AIDS epidemic. The WHO's own scientists concede that it may no longer apply there [32].

Nevertheless, the WHO maintains that where access to DOTS treatment is extensive—that is, not in Africa—its estimated case detection rates are an adequate guide to true TB trends. This is debatable: in China, which is the WHO's finest DOTS success, actual measurements (not estimations) of TB prevalence corroborated the WHO's case detections less well than expected [36].

The best solution now proposed in the scientific literature would redefine the case detection rate, based on measuring true TB prevalence by widespread radiographic or microscopic surveys [31]. Although similar prevalence measurements have been the cornerstone of East Asia's successful attack on TB, the WHO resists changing from estimation to true measurement [37]. As a result, nobody can say with scientific confidence what the actual trends for TB are or whether the TB MDG is on track.

## Child Mortality

The above case studies could leave the dismal impression that all time-limited development goals are immeasurable, lack baseline data, and imply trends having no scientific meaning. Not quite. There is a happy exception: MDG 4, Target 5, which reads to “reduce by two thirds, between 1990 and 2015, the under-five [child] mortality rate” [1].

The under-five child mortality (U5M) rate is an excellent MDG indicator because it is easily measured. For most parents the birth or death of a child is highly memorable; ask properly about these events in a household survey and their recollection is likely to be accurate. If the survey asks enough parents in a population, and continues to ask at regular intervals, a statistically significant trend emerges with time—the very point of the MDGs.

The best proof of this concept comes from Africa. Using data from sequential DHS cycles, in Ghana during 1988–1998, the U5M rate improved 30% [38]. Conversely, in Zimbabwe during 1988–1999, the U5M rate deteriorated 44% [38]. Unlike other MDGs where such changes are, to put it bluntly, only guessed at, these trends in the U5M rate are properly measured and, importantly, are scientifically meaningful, with confidence intervals that reveal the accuracy and quality of the underlying data. Just by keeping the current DHS technique, and interviewing about 7,000 women per country every five years, it is possible to reliably detect either a 15% gain or loss in the U5M rate with scientific confidence.

There is an invaluable and gratifying lesson to draw from the U5M case study: if the UN sets an MDG target that is practical to measure (most are not), and the measurement technique for that MDG target is suitable (most are not), and measurements are taken at the baseline year and in subsequent years (they rarely are), it is then possible to measure the state of the world's health reliably and accurately, and with excellent scientific confidence regarding the trend. In short, it becomes possible to know, not just to guess, if the MDGs are on track or not—even in Africa.

## Discussion

I did not write this paper to doubt the moral necessity of investing more money and political capital in global development; that is unarguable, and it would be reprehensible to use these arguments to seed those doubts.

Instead, I hope to open an important debate, unable to be fully answered by this paper, on a hitherto almost unexplored question: is the world better off with or without the MDGs and similar UN-sponsored, time-limited, quantitative development goals? The answer to that question must be sought without pro-UN or anti-UN ideology, but with awareness that there are two prongs to consider: (i) whether such goals are interpreted so as to advance the dignity and well-being of the large number of people who live in extreme poverty , and (ii) whether such goals advance the reputation of the UN and the global development establishment. I believe the MDGs risk trouble on both fronts.

Viewed objectively, it must be agreed that the MDGs palter. The health goals for 2015 sound quantitative, but for most of them, their quantification is irretrievably flawed. The trends that the health goals allude to are either immeasurable or were not measured properly from the 1990 baseline year onward. This is not an extraordinarily controversial conclusion: recall that in each of the cautionary examples discussed—malaria, maternal mortality, and TB—the UN's own current or former staff have said that the trends are immeasurable or lack baseline data.

Short of abandoning the MDGs, the better option is to amend the goals, targets, or indicators—all three levels of the hierarchy—to be feasibly measurable.

Unfortunately, the UN leadership has, to date, delayed this option. In a September 2004 memo, one year ahead of the Millennium +5 Summit, the UN's Deputy Secretary General instructed the organisation's experts in charge of the MDG statistics with the following:

*The [Millennium +5 Summit]…should not be distracted by arguments over the measurement of the MDGs—or worse, over different numbers being used by different agencies for the same indicator…. [P]roposals for modifications of definitions or new indicators will only be considered formally after the [Millennium +5 Summit]… as any changes at this stage would only distract from the result that we would like to achieve. [3]*

The Deputy Secretary General's order interferes with and shows a profound disrespect for the scientific process—a process that fundamentally is not “distracted by arguments” nor disturbed by “different numbers”. On the contrary, intellectual arguments between scientists are essential for devising new methods of measurements for the MDGs, so that they in turn yield more accurate numbers about the extent and causes of extreme poverty.

By suppressing proposals to amend the MDGs ahead of the Millennium +5 Summit, the UN leadership discarded the only timely opportunity to win high-level political support for truly measurable, scientifically meaningful goals. While the Deputy Secretary General plans “a process that will consider recommendations regarding refinements” to the MDGs, the process will commence only after this September's summit [3]. As a result, any recommendations to amend the MDGs that may arise must await ratification at the next heads-of-state summit—presumably, the Millennium +10 Summit in 2010 (to date, summits occur every five years). In that case, there would remain only five years to the MDGs' final reckoning in 2015. Such extreme delay is illogical and sabotages the MDGs' chances of success.

Some may disagree with my emphasis on measurement and timelines. One anonymous peer reviewer of this paper wrote that while measuring the MDGs is “of concern for epidemiologists and others”, my interpretation “misses the point” because the purpose of the MDGs is merely to be exhortatory. “The MDGs are not a measuring exercise”, wrote the reviewer, but instead are a “common vision of what matters most for improving the lives of people in poor countries”.

This sort of thinking, although widespread among development professionals, is neglectful towards people living in extreme poverty. Neglect occurs when one touts the MDGs for the “common vision” of, say, reducing maternal mortality, while being indolent about measurements to prove mortality is genuinely decreasing. That formulation values consensus about helping pregnant women, ahead of certainty about helping pregnant women—an outcome that, if they knew about it, the women could easily find ideological and dehumanising.

Further, the notion that the MDGs are merely exhortatory discriminates against the world's poorest people. Imagine if European or American leaders, taking aim at poverty in their own countries, set quantitative goals to reduce unemployment or teen pregnancy—only to declare the unemployment and teen pregnancy rates were “not a measuring exercise”. Most people would abhor the dishonesty, for obvious reasons.

But if it is shameful, as I believe, to interpret the MDGs as merely exhortatory, imparting no standards of performance, the converse error also exists: to interpret the MDGs as all-encompassing and imparting too many standards of performance.

The latest fashion, exemplified by the UN Millennium Project, is to treat the MDGs as catch-alls or tautologies for development itself. In a list entitled “Interventions by MDG Target”, the UN Millennium Project recommends to build “roads” or “transport infrastructure” for all of the following MDG targets: primary education, hunger, gender equality, water and sanitation, child mortality, and, of course, malaria, maternal mortality, and TB [39]. Electricity, slum upgrading, and education are similar panaceas.

Definitely roads or electricity matter to holistic development, but justifying those under the cover of goals expressly for child mortality or malaria, makes goal-setting seem pointless. Worse, such justification sounds dishonest—a camouflage job. It is no wonder that with the MDGs subordinated into empty vessels for tenuously related interventions—subordinated into, as Professor Jeffrey Sachs says, just “any international goals”—there is resistance to measure the progress of the specific goals, targets, and indicators with rigor and precision [2].

The MDGs could turn from opportunity to liability.

I believe that without thoroughgoing action to change the current scenario (see Box 2), the MDGs could turn from opportunity to liability. As 2015 nears, the UN becomes increasingly vulnerable to criticism if it still lacks data to prove whether the MDGs are or are not being met. A stream of embarrassing disclosures, similar to the external evaluation of RBM, will likely ensue. Certainly journalists will report the embarrassments, and opponents of foreign aid may use them to discredit further generosity to poor countries. These unhappy events are entirely foreseeable, and for that reason, must give pause to anyone who naively believes that measuring the MDGs is an occupation only scientists need care about. Anyone wishing to preserve the credibility of the UN and the global development enterprise ten years from now also must care.

**Box 2. Five Recommendations to Make the MDGs Truly Time-Limited and Quantitative**

• Convene an external (non-UN) scientific peer review to examine the goals, targets, and indicators to ascertain whether the desired trend of improvement in each is, with current data, measurable or estimable at scientifically accepted levels of accuracy and statistical significance.

• For those goals, targets, or indicators measurable by household surveys, choose only a single survey instrument; determine the minimum sample size needed to detect favourable or adverse trends with statistical significance; conduct the survey at regular intervals; and make all the micro-level data fully public, so independent scientists can replicate the UN's conclusions. Eliminate the many superfluous household surveys now in use.

• For those goals, targets, or indicators not measurable by household surveys, institute sample surveys (“mini censuses”) by creating a large number of new demographic surveillance sites in various countries. The Canadian-funded Tanzania Essential Health Interventions Project is a superb example (see [18]; http://video.idrc.ca/tehip/tehip_dss_e_1000.asx; http://www.economist.com/displaystory.cfm?story_id=1280587).

• For those goals, targets, or indicators that are not measurable by any practical means, first consider to amend them, and if that is not possible, abandon them (bearing in mind that any feasible amendment to the goals, targets, or indicators can only modestly deviate from the political consensus that underpins the MDGs now).

• Within 18 months, hold a high-level UN-sponsored event at which governments ratify final actions for all the above. Have those actions be developed by external scientists and given to the Deputy Secretary General directly.

More thoughtful and timely action for the sake of these institutions, and, needless to say, the millions of people who shall live—or die—with the success or failure of the MDGs, is only wise.

## Abbreviations

DOTS: directly observed therapy—short course
DHS: Demographic and Health Survey
MDG: Millennium Development Goal
MICS: Multiple Indicator Cluster Survey
MMR: maternal mortality ratio
RBM: Roll Back Malaria
TB: tuberculosis
U5M: under-five child mortality
UN: United Nations
UNICEF: United Nations Children's Fund
WHO: World Health Organization
