# Correction: An Immeasurable Crisis? A Criticism of the Millennium Development Goals and Why They Cannot Be Measured

**DOI:** 10.1371/journal.pmed.0030224

**Published:** 2006-05-30

**Authors:** Amir Attaran

In *PLoS Medicine*, volume 2, issue 10: DOI: 10.1371/journal.pmed.0020318

Reference 3 [Deputy Secretary General United Nations (2004) Message to the inter-agency and expert meeting on MDG indicators Geneva 29 September–1 October 2004. New York: United Nations. Available: http://unstats.un.org/unsd/mi/techgroup/Sept2004/message_to_inter_agency_mdg.pdf. Accessed 1 August 2005] was publicly accessible at the time of publication of Amir Attaran's article, but is no longer publicly accessible. This file can now be found as Text S1.

Text S1 Deputy Secretary-General's Message to the Inter-Agency and Expert Meeting on MDG Indicators (44.5 KB DOC).

