# Supplementary material for: Correction: An Immeasurable Crisis? A Criticism of the Millennium Development Goals and Why They Cannot Be Measured
Source: PLoS Med. 2006 May 30;3(5):e224. doi: 10.1371/journal.pmed.0030224 (PMC1468466; doi:10.1371/journal.pmed.0030224)
Supplement: Text S1 — (44.5 KB DOC). [file pmed.0030224.sd001.doc]

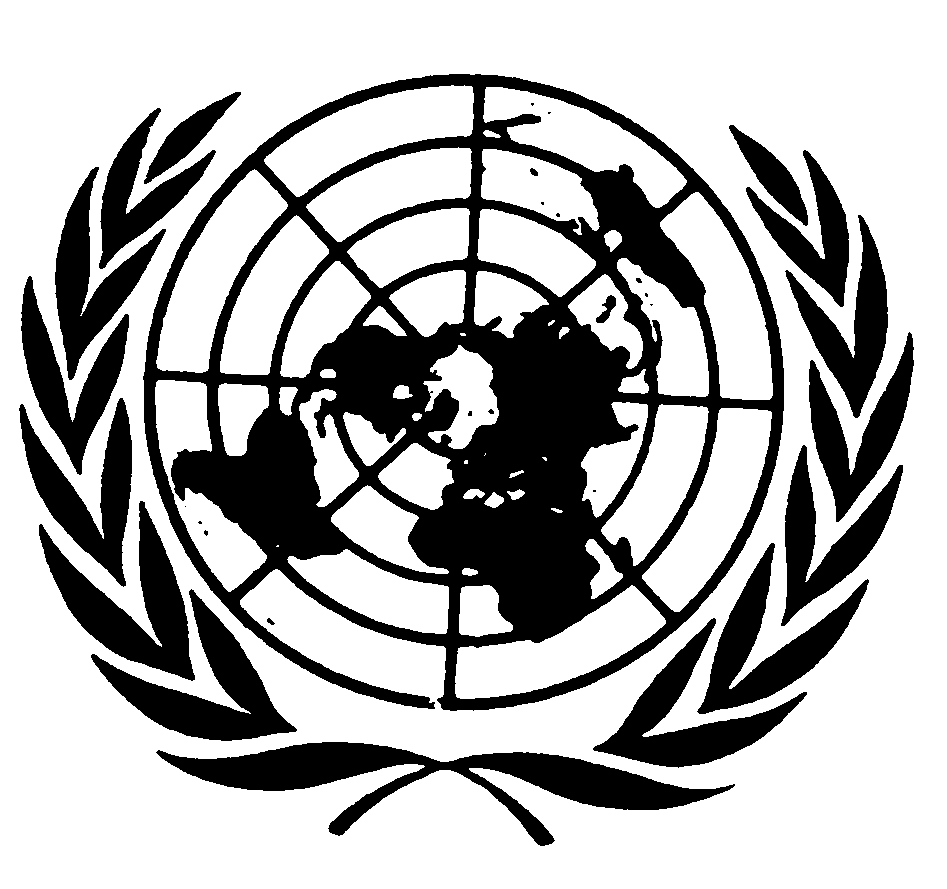


**U N I T E D N A T I O N S N A T I O N S U N I E S**

THE DEPUTY SECRETARY-GENERAL

--

MESSAGE TO THE INTER-AGENCY AND EXPERT MEETING ON MDG INDICATORS

Geneva, 29 September-1 October 2004

*Delivered by Mr. Henk-Jan Brinkman,*

*Senior Economic Affairs Officer, Executive Office of the Secretary-General*

It is my pleasure to welcome you to this meeting as you are embarking on a critical stage in your work.

We have come very far since the adoption of the Millennium Declaration. As the Secretary-General stated in his annual report to the General Assembly, which was released earlier this month, “In four short years, the eight Millennium Development Goals derived from the Millennium Declaration have transformed the face of global development cooperation. The broad global consensus around a set of clear, measurable and time-bound development goals has generated unprecedented, coordinated action, not only within the United Nations system, including the Bretton Woods institutions, but also within the wider donor community and, most importantly, within developing countries themselves.”

I am personally convinced that this is partly a result of your work because statistics are at the core of what the MDGs are all about. Without the carefully defined indicators and the sound quantitative knowledge of how far we have come and the distance we still need to go, we would not have been able to achieve what we have achieved.

As you know, the General Assembly decided to hold a “major event” next year to comprehensively review the Millennium Declaration, including the MDGs. Your work over the next months will be critical to inform the Member States on the progress – and shortfalls – and to provide a solid quantitative foundation for the debates on how to move forward on all the goals. I hope that this major event, which probably will take place in September 2005, will adopt a number of concrete measures that places the world solidly on track to reach the MDGs by 2015.

The negotiations we envisage should not be distracted by arguments over the measurement of the MDGs – or worse, over different numbers being used by different agencies for the same indicator. That is why your agreement on a single, solid set of numbers is so important, especially over the next 12 months.

This also means that proposals for modifications of definitions or new indicators will only be considered formally after the event in 2005, as any changes at this stage would only distract from the result that we would like to achieve. After the event, my office will establish a process that will consider recommendations regarding refinements to the list. That said, I am sure you will have opportunities to discuss these issues in more detail in the coming three days.

Finally, I would like to profoundly thank you for all your hard work and dedication, individually and collectively. I am very much aware of your work and the results it has achieved, and it is being followed personally by me and Robert Orr, the new Assistant Secretary-General assigned to draft the Secretary-General’s Report on the Millennium Declaration, which will be released in March 2005.

I wish you a very successful meeting and I am looking forward to the result of your work at this meeting and over the critical next months.
